# The association between intrinsic motivation and quiet quitting among healthcare workers in Türkiye: a cross-sectional study of the mediating roles of organizational identification and work engagement

**DOI:** 10.3389/fpsyg.2026.1905599

**Published:** 2026-08-13

**Authors:** Haydar Kerem Hoşgör, Derya Gündüz Hoşgör, İbrahim Gün, Salim Yilmaz

**Affiliations:** 1 Medical Services and Technique Department, Vocational School of Health Services, Uşak University, Uşak, Türkiye; 2 Medical Faculty-Trainig and Research Hospital Operating Room, Uşak University, Uşak, Türkiye; 3 Department of Health Management, Faculty of Health Sciences, Batman University, Batman, Türkiye; 4 Department of Healthcare Management, Faculty of Health Sciences, Acibadem Mehmet Ali Aydinlar University, Istanbul, Türkiye; 5 Department of Healthcare Management, Graduate School of Health Sciences, Acibadem Mehmet Ali Aydinlar University, Istanbul, Türkiye

**Keywords:** healthcare workers, intrinsic motivation, organizational identification, quiet quitting, work engagement

## Abstract

**Introduction:**

This study examined the association between intrinsic motivation and quiet quitting among healthcare workers and whether this association was statistically mediated by organizational identification and work engagement.

**Methods:**

Data were collected from 313 hospital employees in Türkiye using an online questionnaire. Intrinsic motivation, organizational identification, work engagement, and quiet quitting were measured using previously validated scales. Mediation analyses were performed using PROCESS Model 4 with 5,000 bootstrap samples. Additional sensitivity analyses were conducted after controlling for profession, weekly working hours, shift type, age, and professional experience.

**Results:**

Intrinsic motivation was positively associated with organizational identification (β = 0.384, *p* < 0.001) and work engagement (β = 0.508, *p* < 0.001), and negatively associated with quiet quitting [total association: β = −0.319, 95% CI (−0.400, −0.235)]. Organizational identification (β = −0.154, *p* = 0.008) and work engagement (β = −0.204, *p* = 0.001) were both negatively associated with quiet quitting. After including the mediators, the direct association between intrinsic motivation and quiet quitting remained statistically significant [β = −0.157, 95% CI (−0.262, −0.032)], indicating statistical partial mediation. Significant indirect associations were observed through organizational identification [β = −0.059, 95% CI (−0.102, −0.012)] and work engagement [β = −0.103, 95% CI (−0.177, −0.022)].

**Discussion:**

Intrinsic motivation was negatively associated with quiet quitting among hospital employees, and this association was statistically partially mediated by organizational identification and work engagement. These findings suggest that employees' psychological attachment to their organization and their engagement with work may represent important mechanisms underlying the observed association.

## 1 Introduction

In recent years, uncertainties in the labor market, economic fluctuations, and increasing workloads have laid the groundwork for a paradigm shift in working life, fundamentally transforming employees' attitudes toward their jobs (Çalişkan, 2023). One of the most notable manifestations of this transformation is the phenomenon of *quiet quitting*. Quiet quitting refers to employees limiting themselves to performing only the duties specified in their job descriptions, withdrawing from organizational citizenship behaviors, and experiencing a decline in their psychological commitment to their work (Kang et al., 2023; Toska et al., 2025). In other words, quiet quitting reflects a tendency among employees to avoid going beyond their prescribed roles, deliberately distance themselves psychologically from work, and confine themselves to only their core responsibilities (Formica and Sfodera, 2022).

The phenomenon of quiet quitting is closely related to the post-pandemic surge in mass employee departures commonly referred to as the “Great Resignation” (Arvaniti et al., 2024). However, some employees, constrained by structural barriers such as economic hardship, financial insecurity, and the inability to find alternative employment, are unable to physically resign; instead, they retract their emotional and cognitive commitment to their workplace, resorting to “quiet quitting” (Uysal and Kim, 2025). From this perspective, quiet quitting does not equate to literal job resignation (Boy and Sürmeli, 2023) and constitutes a limited state wherein employees settle for merely fulfilling basic expectations (Mathushan et al., 2025). Moreover, employees exhibiting this attitude tend to refrain from work-related activities after office hours, avoid responding to incoming emails or phone calls, and prioritize their personal lives (Hetler, 2024). All these tendencies are sometimes associated with passive-aggressive behavior toward the employer (Cohen, 2022), and at other times, they are considered as efforts to establish healthy work boundaries rather than signs of covert or malicious attitudes toward the organization (Cuadra, 2022).

Particularly, the healthcare sector is one of the areas most affected by this trend (Alami et al., 2024). Recent studies have revealed that a significant proportion of healthcare workers exhibit quiet quitting behaviors (Galanis et al., 2024; Moisoglou et al., 2024; Toska et al., 2025). Furthermore, individual factors contributing to quiet quitting include burnout (Gabelaia and Bagociunaite, 2024), low job satisfaction (Galanis et al., 2025), weak motivation, and inadequate job performance (Karalinç, 2024), and stagnation in career development (Lestari et al., 2024). At the organizational level, quiet quitting is associated with decreased service quality (Aiken et al., 2012), reduced productivity, diminished organizational commitment (Ulutürk, 2022), job alienation (Karrani et al., 2025), and an increased turnover intention (Gone et al., 2025). Particularly, healthcare workers performing only the minimum required tasks pose significant risks to patient safety and team collaboration. This situation highlights the need for new managerial strategies (Kang et al., 2023). Additionally, employees exhibiting quiet quitting behaviors may contribute to inadequate care, prolonged hospital stays, unnecessary costs, and unmet patient needs, thereby compromising the efficiency and reputation of healthcare organizations (Xie et al., 2025).

According to the Gallup Global Workplace Report (2023; 2024), quiet quitting not only has individual and organizational repercussions but also affects the global economy. The report indicates that 59% of the global workforce were quiet quitters in 2023, with this figure rising to 62% in 2024. Additionally, 15% of employees reported actively disengaging from their jobs. This disengagement is estimated to cost the global economy approximately $8.9 trillion annually, equating to 9% of global GDP. The global economic cost of this workforce loss is estimated at approximately $8.9 trillion annually. However, in the healthcare sector, this impact is known to be even more detrimental, affecting both healthcare workers and all hospital staff. The aging global population is rapidly increasing the demand for healthcare services. According to the Wilmoth et al. (2023), the number of individuals aged 65 and older was 761 million in 2021, and this figure is projected to rise by at least 110% to reach 1.6 billion by 2050 (Wilmoth et al., 2023). In response to this growing demand, healthcare institutions are grappling with the loss of both healthcare and non-healthcare personnel. Notably, the phenomenon of quiet quitting, which emerged as a significant issue in healthcare institutions during the COVID-19 pandemic, is known to have exacerbated these personnel shortages (Kang et al., 2025).

Quiet quitting is not solely an individual choice but also reflects organizational dynamics and employee psychology (Gürer et al., 2024). In this context, employees' levels of intrinsic motivation emerge as a critical variable in explaining whether they engage in quiet quitting, based on the meaning, value, and satisfaction they attribute to their work. However, this relationship is unlikely to be direct and may instead operate through various psychological and organizational factors. it is believed to be shaped through various psychological and organizational factors. This study examines how two significant variables organizational identification and work engagement mediate the relationship between intrinsic motivation and quiet quitting.

### 1.1 Present study

Employees exhibiting quiet quitting tendencies make no effort to perform beyond their core responsibilities, refrain from undertaking additional duties and avoid exerting extra effort outside regular working hours (Güler, 2023). This situation may lead individuals to perform only at a minimal level in their jobs, rather than leaving positions where they feel unhappy, which can result in a gradual loss of motivation over time (Örücü and Hasirci, 2024). According to Pevec (2023) and Gallup (2023), employees' quiet quitting behavior is a consequence of dissatisfaction or loss of motivation in the workplace. Low motivation levels are reflected in hospital staff's behaviors and can negatively impact the overall structure of healthcare systems (Karaferis et al., 2022). A survey conducted by the ministries of health of multiple countries identified low motivation as the second most significant challenge confronting the healthcare workforce, surpassed only by staff shortages (Mathauer and Imhoff, 2006).

Motivation, defined as the physical or psychological force that drives an individual to engage in behavior, has two primary sources: intrinsic and extrinsic (Robbins and Judge, 2015). Intrinsic motivation refers to the drive to engage in activities due to internal stimuli such as curiosity, the desire to learn, or personal development; whereas extrinsic motivation is influenced by external factors like the pursuit of rewards or the fear of punishment. For instance, a nurse learning new information solely to provide better service to her patients exemplifies intrinsic motivation; whereas adhering to rules to achieve a high score in a performance evaluation illustrates extrinsic motivation. This distinction is supported in the literature by Deci and Ryan's (2013) Self-Determination Theory.

According to Self-Determination Theory, individuals are more likely to regulate their behaviors based on intrinsic interest, curiosity, and personal values, rather than external rewards or pressures, when their basic psychological needs for autonomy, competence, and relatedness are satisfied. Accordingly, intrinsic motivation encourages employees to invest greater psychological resources in their work by enabling them to perceive their jobs not merely as tasks to be completed but as personally meaningful and fulfilling activities (Aydin and Azizoglu, 2022). While intrinsic motivation promotes employees' skill development and professional growth (Garavan et al., 2004), insufficient intrinsic motivation may create conditions conducive to the emergence of quiet quitting behavior (Serenko, 2024). From this perspective, hospital employees with higher levels of intrinsic motivation may be less likely to exhibit quiet quitting attitudes. Given that performing work for enjoyment and personal satisfaction is considered particularly valuable in terms of hospital employees' productivity, the present study focuses exclusively on the intrinsic source of motivation. Previous studies have consistently identified intrinsic motivation as an important antecedent of quiet quitting (Pevec, 2023; Kobak, 2023; Gün, 2024; Agustin and Munawar, 2026; Maheshwari, 2026).

Intrinsic motivation refers to an individual's tendency to perform work voluntarily, with enjoyment, and without the expectation of external rewards (Deci and Ryan, 2013). Employees with high intrinsic motivation demonstrate stronger commitment to their tasks because they perceive their work as meaningful and experience a greater sense of personal value. The literature suggests that intrinsic motivation is one of the major determinants of employees' positive organizational attitudes and behaviors and is positively associated with numerous organizational outcomes, including perceived organizational support (Krishna et al., 2022; Zhu et al., 2025), organizational commitment (Kumar et al., 2016; Fernendes et al., 2024), organizational citizenship behavior (Mandal and Pal, 2025; Abdulmawla et al., 2025), job satisfaction (Ayalew et al., 2021; Al-Sabhan et al., 2022), and psychological empowerment (Meng et al., 2015; Özcan et al., 2025). Hospital employees who are intrinsically motivated tend to evaluate the support they receive from healthcare organizations more positively, derive greater satisfaction from their work, and become more strongly committed to their professional roles. Among these positive organizational outcomes are organizational identification (Rockmann and Ballinger, 2017; Jeong and Han, 2025) and work engagement (Bakar et al., 2019; Zeng et al., 2022).

The sense of meaning and voluntary involvement fostered by intrinsic motivation contributes to hospital employees gradually internalizing the goals and values of their healthcare organizations as part of their own identity. Over time, this process facilitates the integration of employees with organizational values, thereby strengthening organizational identification. In this context, organizational identification is defined as the sense of belonging that hospital employees feel toward their organizations and the psychological bond established between employees and their institutions (Zhi et al., 2024). Organizational identification, defined as employees' perception of themselves as integral members of their organizations, is considered one of the most desirable organizational outcomes, as it fosters employees' willingness to remain with the organization during both favorable and challenging times (Eşkin-Bacaksiz et al., 2017). A review of the literature reveals several studies reporting positive and significant relationships between intrinsic motivation and organizational identification (Özdemir, 2013; Akman, 2017; Rockmann and Ballinger, 2017).

Hospital employees with high intrinsic motivation tend to perceive their work not merely as an obligation but as a meaningful process that contributes to their personal growth (Xu et al., 2022). This perception directly influences the attitudes of healthcare personnel toward their work, encouraging them to approach their jobs with greater energy, attention, and emotional commitment (Özkan et al., 2022). Consequently, when intrinsic motivation is high, hospital employees are naturally more likely to become deeply immersed in their work, maintain concentration, and exert considerable effort. Therefore, intrinsically motivated employees are expected to demonstrate higher levels of work engagement (Siu et al., 2014; Lee and Song, 2020). In this regard, work engagement can be defined as a persistent, positive, affective-motivational state characterized by vigor, dedication, and absorption (Freeney and Fellenz, 2013). Existing studies have consistently reported positive and significant relationships between intrinsic motivation and work engagement (Bakar et al., 2019; Zeng et al., 2022; Aldabbas et al., 2023).

To the best of our knowledge, no previous study has examined the mediating roles of organizational identification and work engagement in the relationship between intrinsic motivation and quiet quitting. Nevertheless, previous research has demonstrated that higher levels of organizational identification and work engagement are associated with lower levels of quiet quitting (Lin and Lin, 2025; Sitorus and Rachmawati, 2024). Based on these findings, it is plausible that hospital employees who increasingly identify themselves with their organizations, successfully integrate with organizational values, and remain highly engaged in their work are more willing to contribute added value to their institutions and invest their efforts in achieving organizational goals. Accordingly, such employees are expected to exhibit relatively lower intentions to engage in quiet quitting. Although empirical evidence remains limited, existing findings suggest that organizational identification and work engagement may represent plausible statistical mediators of the association between intrinsic motivation and quiet quitting.

In light of the aforementioned literature, the aim of this study is to examine the association between intrinsic motivation and quiet quitting, and to reveal the statistical mediating role of organizational identification and work engagement in this relationship. Accordingly, the hypotheses developed within the scope of the research based on the above literature are as follows:

H1a. Intrinsic motivation is positively associated with organizational identification.
H1b. Intrinsic motivation is positively associated with work engagement.
H1c. Intrinsic motivation is negatively associated with quiet quitting.
H2a. Organizational identification is negatively associated with quiet quitting.
H2b. Work engagement is negatively associated with quiet quitting.
H3. Organizational identification would statistically mediate the association between intrinsic motivation and quiet quitting.
H4. Work engagement would statistically mediate the association between intrinsic motivation and quiet quitting.

## 2 Method

This study was designed as a descriptive, cross-sectional study.

### 2.1 Participants

A total of 313 healthcare workers participated in the study. The median age was 34 (±9.88), with 39.6% aged 39 or older. Females comprised 60.7%, and 64.2% were married. The largest proportion of participants held a bachelor's degree (40.3%). Regarding profession, 34.8% were medical secretaries, 24.6% were nurses or midwives, and 16.6% were physicians. The largest proportion worked in administrative units (39.3%), while the majority worked day shifts (58.2%). The majority of participants worked 40 h per week (58.2%). Nearly half of the participants (46.3%) had 11 or more years of professional experience. Detailed participant characteristics are shown in Table 1.

**Table 1:** Socio demographic variables.

Variables	Frequency	Percent
Age (Median age:34 ±9.88)		
19–28 years	91	29.1
29–38	98	31.3
39 and more	124	39.6
Gender		
Female	190	60.7
Male	123	39.3
Marital status		
Single	112	35.8
Married	201	64.2
Education		
High school	29	9.3
Associate degree	91	29.1
Bachelor's degree	126	40.3
Graduate degree	67	21.4
Profession		
Nurse/Midwife	77	24.6
Physician	52	16.6
Health technician/technologist	32	10.2
Medical secretary	109	34.8
Administrative staff	30	9.6
Supportive healthcare staff	13	4.2
Unit of employment		
Internal medicine units	83	26.5
Surgical units	75	24.0
Basic medical sciences units	32	10.2
Administrative units	123	39.3
Type of shift		
Day shift	182	58.2
Duty shift	131	41.9
Weekly working hours		
40	182	58.2
41–48	85	27.2
49 and more	46	14.7
Years of professional experience (years)		
1–5	115	36.7
6–10	53	16.9
11 and more	145	46.3
Total	313	100

### 2.2 Measures

In the present study, a package of questionnaire form, including demographic questions and the following four scales, was used: Intrinsic Motivation Scale, Organizational Identification Scale, Work Engagement Scale, and Quiet Quitting Scale.

#### 2.2.1 Sociodemographic variables

The sociodemographic variables used in the study were as follows: age, gender, marital status, educational level, profession, unit of employment, type of shift, weekly working hours, and years of professional experience.

#### 2.2.2 Intrinsic motivation scale

The Intrinsic Motivation Scale was originally developed by Kuvaas (2006) and Kuvaas and Dysvik (2009). The Turkish adaptation, including the validity and reliability analysis, was conducted by Aydemir Dev et al. (2022). The scale comprises four self-report items rated on a five-point Likert scale (1 = Strongly disagree to 5 = Strongly agree). Higher scores on the scale indicate a higher level of intrinsic motivation. A sample item is “The tasks I perform at work constitute the driving force of my job.” In the Turkish adaptation study, the Cronbach's alpha coefficient was reported as 0.85, whereas in the present study, it was found to be 0.73.

#### 2.2.3 Organizational identification scale

Organizational identification was measured using the six-item Organizational Identification Scale developed by Mael and Ashforth (1992) and adapted into Turkish by Tak Meydan, 2019. The scale consists of six self-report items, each rated on a five-point Likert scale (1 = Strongly disagree to 5 = Strongly agree). Higher scores indicate stronger organizational identification. A sample item is “When someone praises my organization, it feels like a personal compliment.” In the current study, the scale demonstrated good internal consistency, with a Cronbach's alpha coefficient of 0.86.

#### 2.2.4 Ultra-short work engagement scale

The Utrecht Work Engagement Scale (UWES) was developed by Schaufeli et al. (2006) and includes three self-report items assessed on a five-point Likert scale (1 = Strongly disagree to 5 = Strongly agree). The Turkish adaptation and validation of the scale were carried out by Bilginoglu and Yozgat (2019). A sample item is “At my work, I feel bursting with energy.” The original version of the scale demonstrated high internal consistency, with a Cronbach's alpha of 0.93. In the present study, the Cronbach's alpha was calculated as 0.77, indicating acceptable reliability. Higher scores on the scale represent greater levels of work engagement.

#### 2.2.5 Quiet quitting scale

The Quiet Quitting Scale, originally developed by Anand et al. (2023), consists of seven self-report items rated on a five-point Likert scale (1 = Strongly disagree to 5 = Strongly agree). The Turkish validity and reliability study of the scale was conducted by Yildiz et al. (2024). An example item is “I often arrive late and leave early from work.” The scale demonstrated good internal consistency, with a Cronbach's alpha coefficient of 0.829. Higher scores on the scale reflect greater levels of quiet quitting behavior. In our study, the Cronbach's alpha for the scale was 0.78.

### 2.3 Procedure

Prior to commencing the research, permission to use the scales was obtained via email. Subsequently, ethical approval was granted by the Batman University Ethics Committee (REF: 2025/05-05; Decision number: 21). Participants were included in the study after providing voluntary consent. Eligible participants were healthcare workers employed in Türkiye during the data collection period who voluntarily agreed to participate in the study. Participants who did not provide informed consent or submitted incomplete questionnaires were excluded from the analyses. The responses given by the participants were kept confidential, and assurances were provided that the data would remain confidential. Data were collected online in accordance with the Helsinki Declaration. The questionnaire was administered using Google Forms and distributed through social media platforms, including Facebook, X, Instagram, and WhatsApp. Google Forms was configured to accept only one response per participant, thereby minimizing the possibility of duplicate submissions. The survey form used to collect research data consisted of five sections: sociodemographic questions form, Intrinsic Motivation Scale, Organizational Identification Scale, Work Engagement Scale, and Quiet Quitting Scale. Convenience sampling method was employed in the study, and data were collected between June 1 and June 30, 2025. Because the survey link was disseminated through multiple online platforms using convenience sampling, the exact number of individuals who received the invitation could not be determined; therefore, a response rate could not be calculated. Answering all questions in the survey took participants approximately 10 min. To reduce the potential influence of common method bias, several procedural remedies recommended by Podsakoff et al. (2003) were implemented. Participation was anonymous and voluntary, respondents were assured that their responses would remain confidential, and they were informed that there were no right or wrong answers. Furthermore, previously validated measurement instruments were used for all study constructs.

### 2.4 Data analysis

A total of 313 individuals participated in the study. The online survey required responses to all items; therefore, no missing data were present in the final dataset and no imputation was required. The adequacy of the sample size was evaluated according to the recommendations of Fritz and MacKinnon (2007), indicating that the sample size exceeded the suggested range of 115–285 participants required to conduct mediation analysis. Prior to the primary analyses, the characteristics of the scales were assessed, and the normality assumption of the study variables was confirmed. Descriptive statistics (mean and standard deviation) were also calculated. The suitability of the data for normal distribution was tested by examining skewness and kurtosis values in accordance with the methods specified by Tabachnick et al. (2013). Skewness and kurtosis values within the ±2 range indicated that the data were normally distributed. Pearson correlation analysis was conducted to examine the relationships among Intrinsic Motivation, Organizational Identification, Work Engagement, and Quiet Quitting. Subsequently, mediation analysis was performed using PROCESS v4.3 (Model 4). The confidence interval was set at 95%, the significance level accepted at *p* < 0.05, and 5,000 bootstrap samples were used to generate percentile bootstrap confidence intervals (Preacher and Hayes, 2008). PROCESS Model 4 was employed to examine whether the association between the independent and dependent variables was statistically mediated by the proposed mediator variables. The results of the mediation analysis were evaluated by examining the coefficient of determination (*R*^2^), standardized regression coefficients (β), and bootstrap confidence intervals for the indirect effects. These measures provided a comprehensive assessment of the explanatory power of the model and the statistical significance of the indirect effects and the relationships among the study variables (Yildirim et al., 2023). To examine the robustness of the findings, additional mediation analyses were performed by controlling for profession, age, weekly working hours, shift type, and professional experience as covariates. All statistical analyses were performed using SPSS version 25 (IBM Corp., Armonk, NY, USA).

## 3 Results

### 3.1 Preliminary findings

Before testing the hypothesized model, preliminary analyses were conducted, including descriptive statistics, reliability analysis, assessment of normality, and the examination of correlation coefficients. The assumption of normality was evaluated based on the skewness and kurtosis values, using the ±2 threshold recommended by George and Mallery (2010) and Tabachnick et al. (2013). All variables met the normality assumption, as their skewness and kurtosis values ranged between −0.49 and 0.48. Internal consistency was assessed using Cronbach's alpha coefficients, which were found to be acceptable for all constructs: 0.73 for intrinsic motivation, 0.86 for organizational identification, 0.77 for work engagement, and 0.78 for quiet quitting. These results indicate that the scales used in the study demonstrated satisfactory reliability. The results of the correlation analysis are presented in Table 2. Intrinsic motivation was positively and significantly correlated with organizational identification (*r* = 0.384, *p* < 0.01) and work engagement (*r* =.508, *p* < 0.01), while it was negatively correlated with quiet quitting (*r* = – 0.320, *p* < 0.01). Similarly, organizational identification was positively associated with work engagement (*r* =.375, *p* < 0.01) and negatively associated with quiet quitting (*r* = – 0.291, *p* < 0.01). Lastly, work engagement was also negatively and significantly associated with quiet quitting (*r* = – 0.342, *p* < 0.01). The correlation analysis results are presented in Table 2.

**Table 2:** Descriptive statistics and correlations between the study variables.

Variable	Mean	SD	Skew.	Kurt.	α^**^	1	2	3	4
1.Intrinsic motivation	3.51	0.83	−0.39	0.48	0.73	–	0.384^*^	0.508^*^	−0.320^*^
2. Organizational Identification	3.52	0.87	−0.35	−0.26	0.86		–	0.375	−0.291^*^
3. Work engagement	3.50	0.97	−0.49	−0.15	0.77				−0.342^*^
4. Quiet quitting	2.95	0.79	−0.06	−0.23	0.78			–	–

^*^p < 0.05. All correlations are significant at the 0.001 level (two-tailed). SD, standard deviation. Skew., Skewness. Kurt., Kurtosis. α^**^ = Cronbach's alpha reliability coefficient.

### 3.2 Measurement model

Before testing the proposed mediation model, a confirmatory factor analysis (CFA) was conducted to examine the measurement model comprising intrinsic motivation, organizational identification, work engagement, and quiet quitting. The hypothesized four-factor model demonstrated an acceptable fit to the data (χ^2^/df = 2.46, CFI = 0.904, TLI = 0.885, IFI = 0.905, RMSEA = 0.068). Composite reliability (CR), average variance extracted (AVE), and the heterotrait-monotrait ratio (HTMT) were calculated to evaluate construct reliability, convergent validity, and discriminant validity. CR values ranged from 0.714 to 0.832, indicating acceptable internal consistency. Although the AVE values for intrinsic motivation, organizational identification, and quiet quitting were below the conventional threshold of 0.50, the corresponding CR values exceeded 0.70. Therefore, construct validity was evaluated by considering the overall measurement evidence, including composite reliability, discriminant validity (HTMT), and the overall fit of the measurement model. Furthermore, all HTMT values were below the recommended threshold of 0.85, supporting adequate discriminant validity among the study constructs.

### 3.3 Mediation analyses

**Figure 1:**
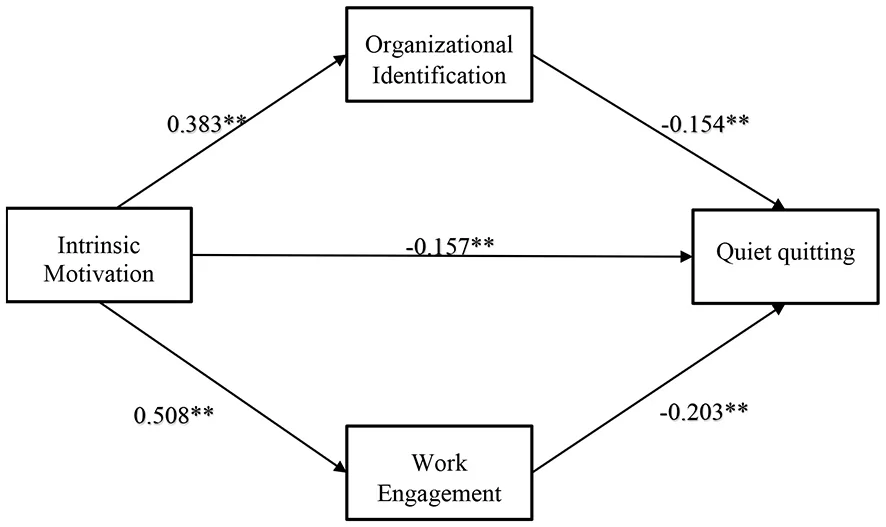
Conceptual model of the hypothesized associations among the study variables. **Standardized coefficient values, *p* < 0.05.

Mediation analyses were carried out to explore the potential mediating roles of organizational identification and work engagement in the relationship between intrinsic motivation and quiet quitting. The findings demonstrated that intrinsic motivation was positively and significantly associated with organizational identification (β = 0.383, *p* < 0.05), supporting H1a, and work engagement (β = 0.508, *p* < 0.05), supporting H1b. Moreover, both organizational identification (β = −0.154, *p* < 0.01), supporting H2a, and work engagement (β = −0.203, *p* < 0.05), supporting H2b, were significantly associated with quiet quitting. Additionally, intrinsic motivation was significantly and negatively associated with quiet quitting (β = −0.157, *p* < 0.05), supporting **H1c** (see Figure 1). The results of the mediation analysis revealed that intrinsic motivation accounted for 14.7% of the variance in organizational identification (*R*^2^ = 0.147, *p* < 0.01) and 25.8% of the variance in work engagement (*R*^2^ = 0.258, *p* < 0.01). Additionally, the full model incorporating intrinsic motivation, organizational identification, and work engagement explained 16.4% of the variance in quiet quitting (*R*^2^ = 0.164, *p* < 0.01; see Table 3).

**Table 3:** Unstandardized regression coefficients for the mediation model.

Antecedent	Consequent											
	M_1_ (Organizational Identification)				M_2_ (Work Engagement)				Y (Quiet Quitting)			
	Coeff.	*SE*	*t*	*p*	Coeff.	*SE*	*t*	*p*	Coeff.	*SE*	*t*	*p*
X (Intrinsic Motivation)	0.400	0.054	7.323	< .001	0.592	0.057	10.407	< 0.001	−0.147	0.058	−2.525	0.012
M_1_ (Orgasizational Identification)									−0.138	0.052	−2.268	0.008
M_2_ (Work Engagement)									−0.164	0.050	−3.290	0.001
Constant									4.534	0.211	21.449	0.000
	*R*^2^ = 0.147				*R*^2^ = 0.258				*R*^2^ = 0.164			
	*F* = 53.630; *p* < 0.01				*F* = 108.318; *p* < 0.01				*F* = 20.327; *p* < 0.01			

Unstandardized regression coefficients (B) obtained from PROCESS Model 4 are reported. Standardized regression coefficients (β) are presented in Table 4 to facilitate interpretation of the direct, indirect, and total associations. Number of bootstrap samples = 5,000. *B*, unstandardized regression coefficient; SE, standard error; M, mediator; X, independent variable; Y, dependent variable.

The mediation analysis indicated that the total association between intrinsic motivation and quiet quitting was significant [β = −0.319, 95% CI (−0.400, −0.235)]. The direct association between intrinsic motivation and quiet quitting remained statistically significant even after including the mediators in the model [β = −0.157, 95% CI (−0.262, −0.032)], suggesting statistical partial mediation. Regarding the indirect associations, intrinsic motivation was found to have a significant negative indirect association with quiet quitting through organizational identification [β = −0.059, 95% CI (−0.102, −0.012)], supporting H3, and work engagement [β = −0.103, 95% CI (−0.177, −0.022)], supporting H4. The total indirect association of intrinsic motivation with quiet quitting through the two mediators was also significant [β = −0.162, 95% CI (−0.231, −0.078)], indicating that these mediators together explain a substantial portion of the relationship. These findings suggest that both organizational identification and work engagement statistically partially mediated the association between intrinsic motivation and quiet quitting (Table 4).

**Table 4:** Direct and indirect effects of intrinsic motivation on quiet quitting.

Effect type	Unstandardized coefficient (*B*)	BootLLCI	BootULCI	Standardized coefficient (β)
Direct effect	−0.147	−0.262	−0.032	−0.157
Indirect effect through				
Organizational Identification	−0.055	−0.102	−0.012	−0.059
Work Engagement	−0.097	−0.177	−0.022	−0.103
Total indirect effect	−0.153	−0.231	−0.078	−0.162
Total effect	−0.300	−0.400	−0.235	−0.319

*B*, unstandardized regression coefficient; β, completely standardized regression coefficient; LLCI, lower limit of the 95% bootstrap confidence interval; ULCI, upper limit of the 95% bootstrap confidence interval. Indirect associations were estimated using 5,000 bootstrap samples.

### 3.4 Sensitivity analysis

As a sensitivity analysis, the mediation model was re-estimated after controlling for profession, weekly working hours, shift type, age, and professional experience. The inclusion of these covariates did not materially change the magnitude or statistical significance of the direct and indirect effects. Intrinsic motivation remained positively associated with organizational identification (β = 0.378, *p* < 0.001) and work engagement (β = 0.513, *p* < 0.001). Furthermore, organizational identification (β = –.155, *p* = 0.009) and work engagement (β = –.184, *p* = 0.004) remained significant mediators of the relationship between intrinsic motivation and quiet quitting. The direct association between intrinsic motivation and quiet quitting also remained significant (β = –.162, *p* = 0.011). Among the covariates, only professional experience was significantly associated with quiet quitting (β = 0.208, *p* = 0.029), whereas the remaining covariates were not statistically significant.

## 4 Discussion

This study examined the statistical mediating roles of organizational identification and work engagement in the association between intrinsic motivation and quiet quitting among 313 hospital employees working in the healthcare sector.

As a result of the study, it was revealed that as the levels of intrinsic motivation among hospital employees increased, their levels of organizational identification and work engagement significantly increased, whereas their levels of quiet quitting significantly decreased. In addition, it was found that as employees' levels of organizational identification and work engagement improved, their tendency toward quiet quitting significantly decreased. Firstly, the positive relationship between intrinsic motivation and the variables of organizational identification and work engagement is highly consistent with Deci and Ryan's Self-Determination Theory. According to this theory, individuals who are intrinsically motivated tend to develop a deeper commitment to and sense of meaning in their work, which in turn leads to the formation of a psychological bond with the organization and a high level of participation in their job. A review of the literature reveals that there are studies reporting positive and significant relationships between intrinsic motivation and both organizational identification (Özdemir, 2013; Akman, 2017; Rockmann and Ballinger, 2017) and work engagement (Bakar et al., 2019; Zeng et al., 2022; Aldabbas et al., 2023). Moreover, the significant decrease in the tendency toward quiet quitting as the level of intrinsic motivation increases indicates that motivation is a critical factor not only for performance but also for organizational sustainability. Considering that quiet quitting is regarded as a sign of employees' withdrawal from their psychological contract with the organization, fostering intrinsic motivation may represent one component of broader organizational strategies aimed at addressing quiet quitting. Another noteworthy finding of the study is the negative relationship between organizational identification and work engagement with the tendency toward quiet quitting. This result indicates that organizational identification strengthens employees' identity-based integration with the organization, while work engagement supports their full physical, cognitive, and emotional involvement in their jobs. Therefore, it can be stated that these two variables not only yield motivational outcomes but also minimize the risk of organizational detachment among employees.

Within the scope of the study, it was concluded that organizational identification and work engagement were significantly explained by intrinsic motivation and significantly associated with quiet quitting. These findings indicate that intrinsically motivated employees develop stronger bonds with the organization and participate in their work at a higher level; in this context, they tend to distance themselves from quiet quitting behaviors. Therefore, it can be stated that the findings obtained in this study are consistent with the results of existing studies in the literature (Siu et al., 2014; Zeng et al., 2022; Xu et al., 2022; Rockmann and Ballinger, 2017; Kobak, 2023). In addition, the findings suggested that organizational identification and work engagement statistically partially mediated the association between intrinsic motivation and quiet quitting. These findings suggest that the association between intrinsic motivation and quiet quitting is statistically partially mediated by employees' organizational identification and work engagement, indicating that employees' psychological bond with their organization and their emotional and cognitive connection to their work are associated with this relationship. The partial nature of the mediation structure suggests that while organizational identification and engagement serve as significant channels in this relationship. The findings suggest that intrinsic motivation is associated with quiet quitting through both direct and indirect pathways. The stronger indirect association observed through work engagement than through organizational identification may reflect the fact that work engagement represents employees' day-to-day psychological investment in their work, whereas organizational identification primarily reflects their psychological attachment to the organization. Because quiet quitting is characterized by reduced discretionary effort and diminished work involvement, work engagement may represent a more proximal psychological mechanism linking intrinsic motivation to quiet quitting. The continued statistical significance of the direct association further suggests that additional organizational and psychological factors may also contribute to this relationship. Notably, the combined indirect association through organizational identification and work engagement was slightly stronger than the direct association, highlighting the importance of these psychological mechanisms in explaining the observed relationship. Within this framework, it is understood that strategies aimed at integrating hospital employees with their organizations and their work should not be limited to enhancing individual motivation; rather, they should include holistic policies that foster a sense of belonging and deepen the meaningful connection employees establish with their work. Furthermore, the absence of studies addressing the mediating role of organizational identification and work engagement in the relationship between intrinsic motivation and quiet quitting complicates the interpretation of the obtained results, while simultaneously contributing to filling the gaps in the existing literature. At the same time, quiet quitting is a multifaceted organizational phenomenon that cannot be explained solely by intrinsic motivation, organizational identification, and work engagement. Other factors, such as burnout (Hoşgör and Gün, 2026), job satisfaction (Galanis et al., 2025), workload (Taşkesen et al., 2025), leadership practices (Mannan et al., 2026), organizational justice (Kang et al., 2025), institutional support (Singh et al., 2025), and working conditions (Moisoglou et al., 2025), may also contribute to employees' quiet quitting tendencies and partly explain the remaining unexplained variance in the model. Therefore, the present findings should be interpreted within the broader organizational context rather than suggesting that enhancing intrinsic motivation alone is sufficient to reduce quiet quitting. From a practical perspective, interventions designed to support employees' intrinsic motivation are likely to be more effective when combined with organizational initiatives that improve leadership quality, fairness, institutional support, and working conditions. Finally, the robustness of the findings was further supported by sensitivity analyses showing that the observed associations remained statistically significant after controlling for key demographic and occupational characteristics.

## 5 Implications and limitations

The findings of this study suggest that intrinsic motivation is negatively associated with quiet quitting among hospital employees and that this association is statistically partially mediated by organizational identification and work engagement. The obtained results provide important practical implications for healthcare managers aiming to enhance employee commitment and internal organizational stability. Specifically, work environments that support intrinsic motivation may foster employees' organizational identification and work engagement, which, according to the proposed model, are statistically associated with lower levels of quiet quitting. In this context, practices that support employees' basic psychological needs; including autonomy, competence, and relatedness may represent one component of broader organizational strategies aimed at promoting employee engagement and organizational sustainability. The observed negative associations between organizational identification, work engagement, and quiet quitting suggest that healthcare institutions may benefit from initiatives designed to strengthen employees' sense of organizational belonging and engagement at work. For example, ensuring the alignment of employees' values with organizational values, providing meaningful work experiences, and adopting leadership approaches that support psychological capital can contribute to this process. These initiatives should be implemented alongside broader organizational efforts to improve working conditions, fairness, institutional support, and leadership practices. Accordingly, the findings may provide useful insights for hospital managers when developing human resource practices aimed at fostering employee motivation, organizational identification, and work engagement. Nevertheless, enhancing intrinsic motivation alone is unlikely to fully address quiet quitting unless it is accompanied by broader organizational improvements in leadership, workload management, organizational justice, institutional support, and working conditions. Although this study provides important findings regarding the relationships among the variables, its cross-sectional design presents limitations in determining causal relationships. Accordingly, the observed mediation should be interpreted as statistical mediation within the proposed theoretical model rather than as evidence of temporal or causal mediation. The cross-sectional design does not establish temporal precedence, and alternative directional relationships among the study variables cannot be ruled out. Therefore, future longitudinal studies are needed to examine the temporal ordering of the observed associations, while experimental studies may help clarify the potential causal relationships among the study variables. Such methodological approaches will enhance the validity and generalizability of the findings. Additionally, studies incorporating other variables such as extrinsic motivation, leadership styles, perceived organizational justice, and support would increase conceptual richness. The findings should be interpreted with caution, as the data were collected through an online survey among healthcare workers in Türkiye, which may limit the generalizability of the results. Studies conducted in different cultural and organizational contexts are important for testing the generalizability of the findings (Yilmaz and Cene, 2026). Responses provided by participants through self-report surveys may be subject to systematic biases such as social desirability bias. Although several procedural and statistical measures were implemented to reduce the potential influence of common method bias, the possibility of residual common method variance cannot be completely excluded. Accordingly, qualitative research exploring employees' perceptions of quiet quitting in depth may contribute to a better understanding of the subject.

## 6 Conclusions

This study revealed that the tendency toward quiet quitting among individuals working in the healthcare sector is closely related to levels of intrinsic motivation, organizational identification, and work engagement. The findings demonstrated that intrinsically motivated employees develop a stronger emotional bond with their organizations and exhibit higher levels of psychological involvement in their work. This factor emerges as a significant determinant in reducing quiet quitting behaviors. The research showed that organizational identification and work engagement are not only associated with intrinsic motivation but also play a decisive role in quiet quitting, serving as partial mediators in the relationship between intrinsic motivation and quiet quitting. This finding highlights the necessity for organizations to develop multidimensional strategies that enhance not only individual motivation but also organizational commitment and work engagement. Consequently, in preventing employees' quiet quitting behaviors, policies that simultaneously support intrinsic motivation at the individual level and strengthen belongingness and commitment at the organizational level must be addressed together. This approach is critically important for healthcare institutions both to improve service quality and to reduce employee turnover rates.

## Data Availability

The raw data supporting the conclusions of this article will be made available by the authors, without undue reservation.
